# Medullary thyroid carcinoma and duodenal calcitonin-secreting neuroendocrine tumour: more than coincidence?

**DOI:** 10.1530/EDM-13-0021

**Published:** 2013-07-15

**Authors:** I Huguet, C Lamas, R Vera, A Lomas, R P Quilez, A Grossman, F Botella

**Affiliations:** 1 Departments of EndocrinologyUniversity Hospital ComplexAlbaceteSpain; 2 PathologyUniversity Hospital ComplexAlbaceteSpain; 3 OCDEM, Churchill HospitalOxfordUK

## Abstract

Neuroendocrine tumours (NETs) are a heterogeneous group of neoplasms whose management can be problematic. In many cases, multiple tumours may occur in the same patient or his or her family, and some of these have now been defined genetically, although in other cases the underlying gene or genes involved remain unclear. We describe a patient, a 63-year-old female, who was diagnosed with a medullary thyroid carcinoma (MTC), which was confirmed pathologically after thyroidectomy, but whose circulating calcitonin levels remained elevated after thyroidectomy with no evidence of metastatic disease. Subsequently, an entirely separate and discrete duodenal NET was identified; this was 2.8 cm in diameter and was removed at partial duodenectomy. The tumour stained immunohistochemically for calcitonin, and its removal led to persistent normalisation of the circulating calcitonin levels. There was no germline mutation of the *RET* oncogene. This is the first identification of a duodenal NET secreting calcitonin and also the first demonstration of a second tumour secreting calcitonin in a patient with MTC. We suggest that where calcitonin levels remain high after removal of a MTC a search for other NETs should be conducted.

**Learning points:**

- NETs are a complex and heterogeneous group of related neoplasms, and multiple tumours may occur in the same patient.
- Calcitonin can be produced ectopically by several tumours outside the thyroid.
- Persistently elevated calcitonin levels after removal of a MTC may not necessarily indicate persisting or metastatic disease from the tumour.
- The real prevalence of calcitonin-producing NETs may be underestimated, as serum determination is only recommended in the diagnosis of pancreatic NETs.

## Background

Despite an increase in prevalence in recent years, neuroendocrine tumours (NETs) are still a relatively rare finding and a clinical challenge because of their low incidence (2–5′/100 000 cases per year (1) (2)), the heterogeneity of this group of tumours and the variability in their clinical behaviour and prognosis.

Duodenal NETs constitute some 4% of all carcinoid neoplasias and 1–3% of all duodenal tumours (1). Although they can potentially produce a wide variety of systemic and locoregional symptoms (anaemia, intestine obstruction and diarrhoea), only 10% are associated with a functional syndrome, and so in most of the cases, the patient remains asymptomatic until the mass effect dominates or they are discovered as an incidental finding at endoscopy or surgery (3) (4). It has been estimated that it usually takes 7 years from the appearance of the first symptom until the diagnosis of a mid-gut carcinoid (5).

Medullary thyroid carcinoma (MTC) is a NET of thyroid parafollicular cells, with some 20% showing germline mutations of the *RET* oncogene. It often appears as a single thyroid nodule located in the upper two-thirds of a lobe, reflecting the anatomical location of the parafollicular cells.

We report the association of two co-existing NETs, a MTC and an extremely rare calcitonin-secreting duodenal tumour, complicating the follow-up of the patient in whom we suspected persisting or metastatic disease.

## Case presentation

**Figure 1:**
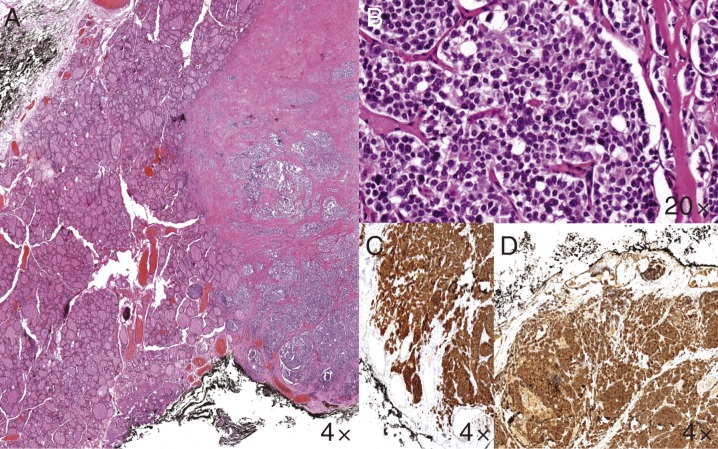
Medullary thyroid carcinoma. (A) Malignant neoplasia infiltrating normal thyroid tissue. (B) Monomorphic uniform nuclei cells, granular chromatin pattern, eosinophilic cytoplasm and little mitosis. HE, 20×.

A 63-year-old woman was referred to our clinic following the incidental finding of a 1 cm thyroid nodule. Fine-needle aspiration cytology revealed a MTC. Subsequently, plasma calcitonin levels were found to be elevated at 84 pg/ml (normal <11.5 pg/ml). There were no other abnormal findings. The presence of a co-existing phaeochromocytoma was biochemically excluded (normal urinary catecholamine and metanephrine levels), and the patient was subjected to total thyroidectomy with clearance of central and lateral lymph node compartments. The pathology demonstrated a calcified 1 cm nodule consisting of polygonal cells showing positive immunostaining for chromogranin, calcitonin, S-100 and carcinoembryonic antigen (CEA; Fig. 1). The lymph nodes were clear of disease. Genetic analysis of peripheral lymphocytes of the *RET* oncogene (automated sequencing of the flanking exons 10, 11, 13, 14, 15 and 16) did not reveal any germline mutation.

## Investigation

The patient recovered well from the operation, but exhibited persistently elevated plasma calcitonin levels, although she remained asymptomatic. Over the following 3 years, her plasma calcitonin levels were persistently elevated, although with no clear signs of progression (106, 116, 83, 173, 212, 279 and 114 pg/ml). Her circulating CEA levels remained normal. We suspected persisting or metastatic disease, but further repeated and detailed imaging including computed tomography (CT) and magnetic resonance (MR) scanning of the neck, chest and abdomen failed to reveal any evidence of tumour. Functional imaging with radiolabelled octreotide (Octreoscan) and fluorodeoxyglucose (FDG)-positron emission tomography (PET) scanning demonstrated mild uptake of both tracers in the midline, adjacent to L2, and further CT scanning was undertaken concentrating on this area. No clear abnormality was observed, and it was concluded at this stage that the apparent uptake was due to duodenal ‘physiological tracer elimination’.

However, after 3 years, the patient was found to have developed an iron-deficiency anaemia associated with positive faecal occult blood testing. Endoscopy was undertaken, and it showed chronic atrophic gastritis with intestinal metaplasia, but in addition a large (3 cm) polyp was found in the second part of the duodenum, which was biopsied.

## Treatment

**Figure 2:**
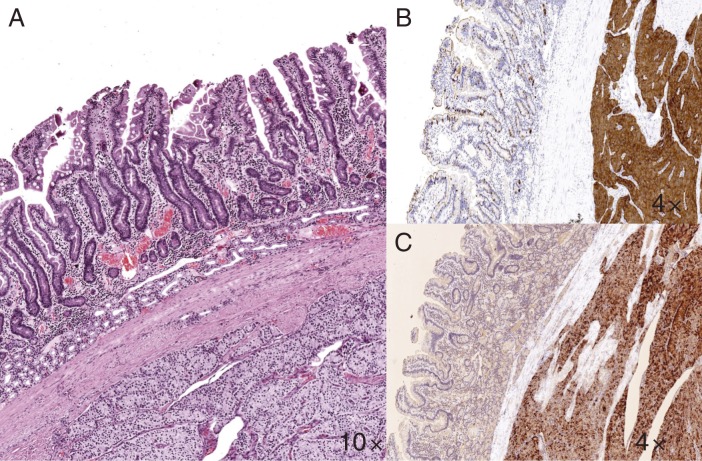
Duodenal tumour. (A) Neuroendocrine neoplasia, mild-to-moderate atypia, irregular cell growth pattern that affects mucosa and submucosa and vascular invasion. HE, 20×. Positive immunohistochemistry for calcitonin (B) and synaptophysin (C).

The duodenal biopsy showed a NET, and the patient was subsequently re-explored surgically and partial duodenectomy was performed. Pathological examination confirmed a 2.8 cm well-differentiated NET with positive immunohistochemistry for CAM5.2, ac1-AE3, enolase, chromogranin, synaptophysin and serotonin; the Ki-67 index was <2% (grade 1). Surprisingly, after resection of the duodenal tumour, circulating calcitonin levels remained repeatedly undetectable, and thus the tissue was immunostained for calcitonin; this was strongly positive (Fig. 2).

## Outcome and follow-up

**Figure 3:**
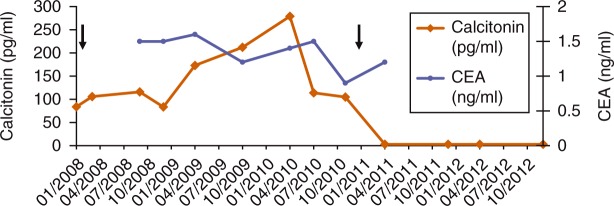
Calcitonin and CEA levels. The first arrow indicates the date of the thyroid surgery (Feb/2008). The second arrow indicates the date of the duodenal surgery (Feb/2010).

Currently, the patient remains asymptomatic with persistently undetectable serum calcitonin levels and no further anaemia. She remains with mildly elevated serum chromogranin A and gastrin levels (last determination of serum gastrin levels: 591 pg/ml, normal <40; Fig. 3), which we attribute to her chronic atrophic gastritis.

## Discussion

We have reported a case of the association of two NETs identified in one patient, both associated with calcitonin secretion, but in the absence of a germline *RET* mutation.

Calcitonin is a peptide hormone that is normally secreted by thyroid C cells, but may be produced ectopically by pancreatic NETs, phaeochromocytomas, melanomas, cervical cancer, breast and colorectal cancers, and small-cell lung and other pulmonary cancers, but usually in association with other ectopically produced peptides (6). In a prospective study, Fleury *et al*. (7) systematically determined calcitonin levels in 66 patients with pancreatic NETs referred to their service over 3 months: elevated levels were found in six patients (9% of the total). While calcitonin is currently recognised as a tumour marker for NETs, its routine determination is only recommended at diagnosis for pancreatic NETs (7).

For the follow-up of MTC, the determination of circulating calcitonin levels is of central importance. After total thyroidectomy, detectable calcitonin values are strongly indicative of residual disease or possible recurrence or metastasis, and changes of this marker in the serum can be observed much earlier than changes in imaging. However, in this case, we were surprised by the continuing high levels of calcitonin as there appeared to have been total clearance of the original small MTC.

The American Thyroid Association guidelines recommend that post-operative patients with plasma calcitonin levels >150 pg/ml should undergo neck ultrasound and additional imaging techniques for the evaluation of distant metastases (grade: B recommendation) (8). Optional imaging techniques include cervico-thoracic CT, liver multislice CT or MR imaging (MRI) with contrast, MRI of the spine and pelvis, and isotopic bone scanning. Recently, ^68^gallium DOTA-octreotide (^68^Ga DOTATOC) has shown better visualisation of small lesions compared with the ^111^In-labelled counterpart in NETs, but despite some studies being carried out using ^68^Ga-labelled DOTA-lanreotide in MTC in order to establish the clinical value and obtain the best imaging protocol of this new tracer, further studies have to be conducted (9). ^18F^18-FDG)-PET or ^18^F-Dopamine (^18^F-DOPA) scans may also be useful, but the sensitivity of these scans is greatly diminished when calcitonin levels are <1000 pg/ml (10). Metaiodobenzylguanidine (MIBG) uptake in MTC is unpredictable, and the data about the use of MIBG show that only a small fraction (about 30%) of MTCs are able to concentrate it, but it can be used as a complement for the rest of the techniques in a combined diagnostic approach (11). In our patient, the standard cross-sectional imaging with CT and MR was unhelpful, and we proceeded to use functional imaging. However, the uptake observed in both the octreotide scan and the FDG-PET scan was misinterpreted as physiological as there was no clear cross-sectional correlate.

This appears to be a unique case of a patient with a duodenal tumour secreting calcitonin, which has not been described previously in a patient with a previous classical MTC. While we regard this association as more than coincidental, we cannot at present identify any genetic predisposition.

## Patient consent

The authors confirm that informed consent has been obtained from the patient for publication of this article and accompanying images.
